# Genomic analysis of Spanish wheat landraces reveals their variability and potential for breeding

**DOI:** 10.1186/s12864-020-6536-x

**Published:** 2020-02-04

**Authors:** Laura Pascual, Magdalena Ruiz, Matilde López-Fernández, Helena Pérez-Peña, Elena Benavente, José Francisco Vázquez, Carolina Sansaloni, Patricia Giraldo

**Affiliations:** 1 Department of Biotechnology-Plant Biology, School of Agricultural, Food and Biosystems Engineering, Universidad Politécnica de Madrid, Madrid, Spain; 2 National Plant Genetic Resources Centre, National Institute for Agricultural and Food Research and Technology, Alcalá de Henares, Spain; 3 Centro Internacional de Mejoramiento de Maíz y Trigo (CIMMYT), Texcoco, Mexico

**Keywords:** Wheat improvement, Local germplasm, GBS, DArTseq markers, SNP, Genetic diversity, Population structure

## Abstract

**Background:**

One of the main goals of the plant breeding in the twenty-first century is the development of crop cultivars that can maintain current yields in unfavorable environments. Landraces that have been grown under varying local conditions include genetic diversity that will be essential to achieve this objective. The Center of Plant Genetic Resources of the Spanish Institute for Agriculture Research maintains a broad collection of wheat landraces. These accessions, which are locally adapted to diverse eco-climatic conditions, represent highly valuable materials for breeding. However, their efficient use requires an exhaustive genetic characterization. The overall aim of this study was to assess the diversity and population structure of a selected set of 380 Spanish landraces and 52 reference varieties of bread and durum wheat by high-throughput genotyping.

**Results:**

The DArTseq GBS approach generated 10 K SNPs and 40 K high-quality DArT markers, which were located against the currently available bread and durum wheat reference genomes. The markers with known locations were distributed across all chromosomes with relatively well-balanced genome-wide coverage. The genetic analysis showed that the Spanish wheat landraces were clustered in different groups, thus representing genetic pools providing a range of allelic variation. The subspecies had a major impact on the population structure of the durum wheat landraces, with three distinct clusters that corresponded to subsp. *durum*, *turgidum* and *dicoccon* being identified. The population structure of bread wheat landraces was mainly biased by geographic origin.

**Conclusions:**

The results showed broader genetic diversity in the landraces compared to a reference set that included commercial varieties, and higher divergence between the landraces and the reference set in durum wheat than in bread wheat. The analyses revealed genomic regions whose patterns of variation were markedly different in the landraces and reference varieties, indicating loci that have been under selection during crop improvement, which could help to target breeding efforts. The results obtained from this work will provide a basis for future genome-wide association studies.

## Background

Wheat is a cereal that belongs to the Poaceae family. Wheat occupies a central place in human nutrition providing 20% of the daily protein and food calories of the human population. Currently cultivated wheat originated from natural hybridization events between different species [1]. Roughly 90 to 95% of the wheat produced in the world is common, or bread wheat (*Triticum aestivum* L.; 2n = 6x = 42, 17Gb, AABBDD genomes). The remainder of the world’s wheat production includes about 35–40 million tons of durum wheat (*T. turgidum* var. *durum*; 2n = 4x = 28, 13 Gb, AABB genomes), which is cultivated mainly in the Mediterranean region (http://www.fao.org/faostat/en/).

Advances in molecular biology and high-throughput genotyping technologies have significantly impacted the field of molecular plant breeding, leading to shift from a phenotype-based to a genotype-based selection [2]. The integrated use of genomic and molecular tools in conventional phenotype selection programs has allowed the development of new breeding strategies such as marker assisted selection (MAS) and genomic selection. However, in wheat, the large complex genome with an over 85% repetitive DNA content has hampered the application of these molecular breeding approaches compared to their use in other crops, as the presence of two or three separate but closely related subgenomes hinders the analysis of homoeologous gene sequences [3]. The recently published durum and bread wheat reference genomes [4, 5] provide high-quality data that will help to physically locate thousands of scattered molecular markers, thus facilitating the identification of key genes by genome-wide association studies (GWAS) that will be highly valuable for MAS in wheat breeding programs [6].

The successful genomics-assisted breeding of any crop will be enhanced by a thorough understanding of the species’ genetic diversity. As is the case in other crops, genetic diversity of wheat has declined as a consequence of bottlenecks encountered during polyploidization and domestication [7, 8]. Modern plant breeding practices, in which only a small number of elite cultivars are included in crossing programs, have further narrowed the genetic base of wheat throughout the world, limiting the pool of alleles in which to search for new traits of agronomic interest. This has promoted wide crossing programs carried out since the 1980s at different centers of wheat research such as CIMMYT (Centro Internacional de Mejoramiento de Maíz y Trigo). Indeed, by 1990, CIMMYT breeders began to successfully increase wheat productivity and genetic diversity through the introgression of various novel wheat materials. However, the genetic diversity represented by current wheat cultivars needs to be further increased to face novel threats, such as climate change, which demands an enlarged pool of alleles. Fortunately, an enormous number of genetically different, locally well-adapted wheat landraces were generated through natural or farmer-mediated selection in the previous century. Because future gains in yield potential will surely require the exploitation of these largely untapped sources of genetic diversity [9], deep knowledge of their genetic/genomic diversity is highly valuable to address the forthcoming plant breeding challenges [10].

A large number of studies have been performed to estimate genetic diversity by employing different methodologies in diverse plant species [11, 12], including wheat [13]. It is accepted that molecular markers are the best option for genetic variation studies. Among these markers, single nucleotide polymorphisms (SNPs), whose detection has been enormously facilitated by high-throughput technologies such as SNP arrays [14] or genotyping-by-sequencing (GBS) [15], are the most frequently used for genome-wide diversity studies.

The assessment of genome-wide diversity by GBS provides robust estimates of diversity and has been increasingly adopted as a fast, high-throughput cost-effective tool for whole-genome genetic diversity analysis in large germplasm sets [16]. The DArTseq (Diversity Array Technology sequence) markers, based on GBS [17], efficiently target low-copy-number sequences via a complexity reduction method and have been successfully applied for genetic diversity studies in different species [18–21]. Moreover, DArTseq provides data at an affordable cost, especially in complex polyploid species such as wheat (https://www.diversityarrays.com), where it has been extensively used [20, 22, 23]. It is indeed the method employed by CIMMYT to build the most comprehensive genotype datasets for genetic resources in wheat (https://seedsofdiscovery.org/about/genotyping-platform/).

High-throughput genotyping also provides essential information for the design of high-power GWAS, which enable the identification of agriculturally important genes and facilitate their transfer from wild or local germplasm into modern cultivars through marker-assisted selection and marker-assisted breeding and/or genomic selection. For GWAS analysis, the optimum diverse panel must be genotyped with a set of molecular markers covering as much of the genome of the species as possible [24], but the population structure needs to be investigated to avoid false associations between phenotypes and markers [25].

The Spanish wheat landraces conserved at the National Plant Genetic Resources Center (CRF-INIA) and maintained in the national collection were collected in the first half of the twentieth century. Several studies have shown the great variability of the Spanish durum wheat accessions compared to other germplasm collections [26–29]. However, no genetic description of the bread wheat landraces has been reported, and the high-throughput genomic characterization of the durum wheat landraces remains to be fully realized.

The aim of the present study was to characterize two collections of durum and bread wheat landraces from CRF-INIA by using the DArTseq-GBS approach. The specific objectives of the present investigation were: (1) to assess the genomic diversity of a set of durum wheat accessions comprising 191 Spanish landraces and 23 reference varieties, (2) to assess the genomic diversity of a set of bread wheat accessions comprising 189 Spanish landraces and 29 reference varieties, and (3) to compare the genetic diversity of landraces and modern cultivars in both wheat species.

## Results

### Wheat genotyping

We characterized a set of 380 landraces and 52 reference varieties at genomic level (Additional file 1). The DArTseq approach allowed us to detect approximately 100 K DArTs (presence/absence markers) and 50 K SNPs in each analyzed species.

In tetraploid durum wheat (214 accessions), a total of 98,983 DArTseq markers and 51,751 SNP markers were obtained (Additional files 2 and 3). When the markers were located in the *T. turgidum* reference genome, they were distributed throughout the genome (Table 1). According to the raw data, approximately 58% of the DArTs, and 37% of the SNPs were not located in the *T. turgidum* reference genome. After filtering to obtain highly informative markers, 38,700 DArTs and 9324 SNPs were selected for further analysis. In this set of markers, the percentage of located markers was similar to that from the raw data (66% in SNPs and 45% in DArTs). As shown in Table 1, the filters applied did not affect the marker distribution within the genome. The A and B genomes presented a comparable number of markers, both before and after filtering. Chromosome 4B exhibited the lowest density of both types of markers.

**Table 1:** Numbers of SNP and DArT markers identified in *T. turgidum* and *T. aestivum* accessions. The total numbers of markers before and after filtering, and their distribution within the genomes and chromosomes are presented. NA, no data available, as D genome is not present in *T. turgidum*

	*Triticum turgidum*				*Triticum aestivum*			
	Raw DARTs	Filtered DARTs	Raw SNPs	Filtered SNPs	Raw DARTs	Filtered DARTs	Raw SNPs	Filtered SNPs
Total	98,983	38,700	51,751	9324	130,899	44,241	58,660	8238
Located	41,429	17,442	32,811	6192	46,665	16,090	34,497	4738
Not located	57,554	21,258	18,940	3132	84,234	28,151	24,163	3500
Genome A	19,307	7907	15,719	2957	16,127	5957	12,762	1958
Genome B	22,122	9535	17,092	3235	17,754	7000	13,636	1963
Genome D	NA	NA	NA	NA	12,784	3133	8099	817
1A	2047	863	1758	378	1841	723	1594	285
1B	2972	1311	2341	505	2431	952	1888	278
1D	NA	NA	NA	NA	1797	384	1046	101
2A	3079	1173	2581	519	2549	891	2072	302
2B	4017	1661	3117	553	3188	1272	2500	345
2D	NA	NA	NA	NA	2501	773	1627	156
3A	2757	1057	2252	409	2258	687	1923	316
3B	3604	1570	2795	527	2763	1076	2256	331
3D	NA	NA	NA	NA	1877	406	1279	106
4A	2587	1050	1805	295	2233	877	1453	167
4B	1763	743	1328	295	1492	491	1028	145
4D	NA	NA	NA	NA	1033	170	515	71
5A	2603	998	2224	486	2135	701	1749	330
5B	3054	1197	2332	462	2572	1007	1948	299
5D	NA	NA	NA	NA	1731	383	1068	119
6A	2364	1027	1819	306	1909	777	1385	203
6B	3360	1541	2567	411	2467	1027	1970	283
6D	NA	NA	NA	NA	1536	432	1013	118
7A	3870	1739	3280	564	3202	1301	2586	355
7B	3352	1512	2612	482	2841	1175	2046	282
7D	NA	NA	NA	NA	2309	585	1551	146

For hexaploid bread wheat (218 accessions), a slightly higher number of markers, including 130,899 DArTseq markers and 58,660 SNPs, was generated (Additional files 4 and 5). As in durum wheat, the markers were detected throughout the whole genome; around 64% of raw DArTs and 41% of raw SNPs were not located in the bread wheat reference genome, a percentage similar to that of the durum wheat (Table 1). After filtering, 44,241 DArTs and 8238 SNPs were selected for further analysis. In this set of markers, the percentage of located markers was similar to the percentage obtained in the raw data (36% in DArTs and 57% in SNPs). The D genome presented a reduced amount of markers compared to A and B genomes. Regarding these latter, chromosome 4B exhibited again the lowest density of both types of markers.

**Fig. 1:**
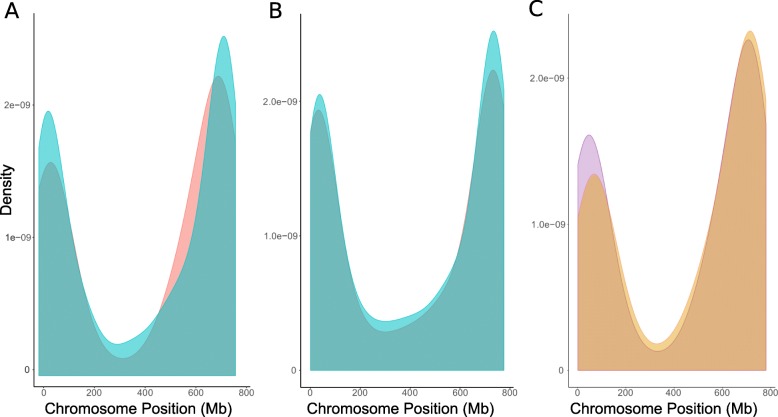
Marker density along chromosome 2A. **a** *T. turgidum* raw (blue) and filtered (red) SNP markers. **b** *T. turgidum* raw (blue) and filtered (red) DArT markers. **c** Filtered SNPs in *T. turgidum* (purple) and *T. aestivum* (yellow)

The marker distribution along the chromosomes was comparable in bread and durum wheat for all the A and B genome chromosomes. In general, a higher density of markers was found at both chromosome ends, as illustrated for chromosome 2A in Fig. 1. The same pattern was observed for the bread wheat D genome chromosomes.

**Fig. 2:**
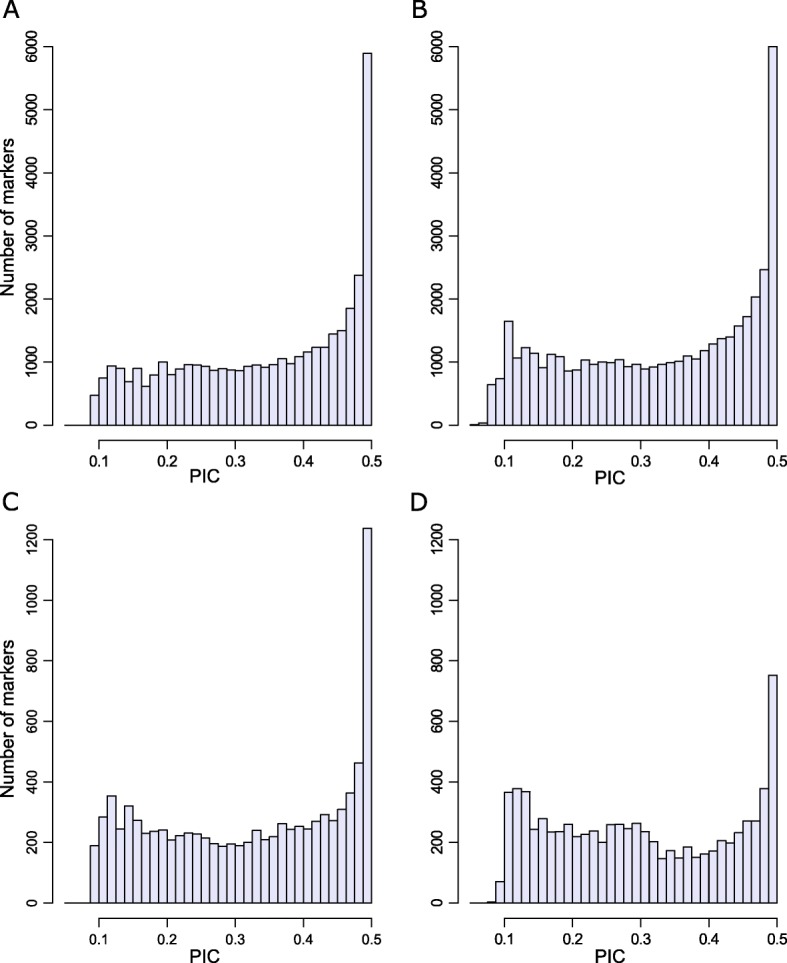
Average PIC distribution in filtered markers. **a** *T. turgidum* DArTs. **b** *T. aestivum* DArTs. **c** *T. turgidum* SNPs. **d** *T. aestivum* SNPs

In both species, the distribution of PIC (polymorphic index content) values for the DArT and SNP data was asymmetrical and skewed towards the lower values (Fig. 2). In durum wheat, 82% of the DArTs and 75% of the SNP markers showed a PIC value > 0.2. In bread wheat, the corresponding values were 76 and 70% for DArTs and SNPs, respectively. For both species and types of markers, the average PIC values were between 0.30 and 0.35.

### Genetic structure of the durum wheat collection

**Fig. 3:**
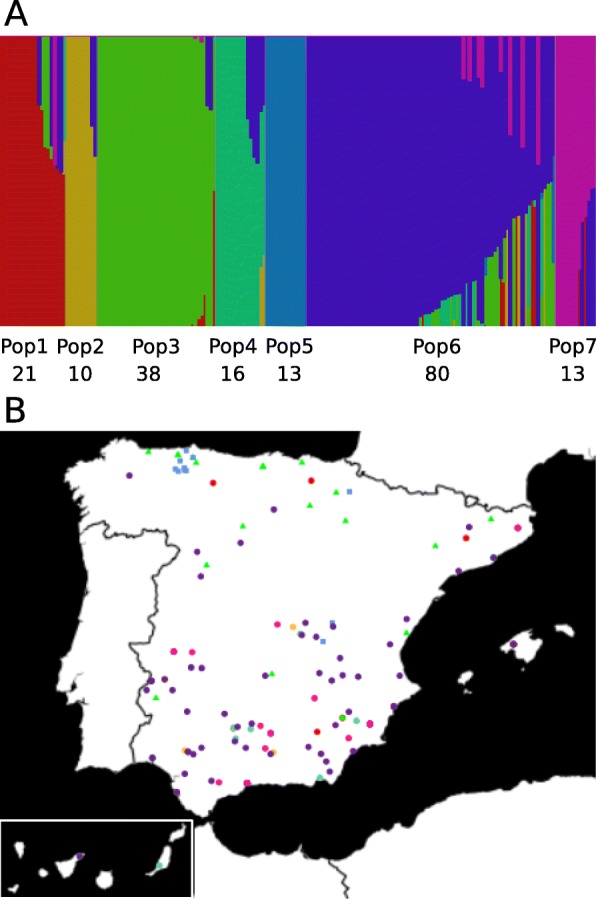
**a** *T. turgidum* STRUCTURE plot based on DArT markers. The number below the Pop indicates the number of accessions clustered in each population. **b** Collection sites of the different *T. turgidum* accessions, colored according to their STRUCTURE population assignment. When GPS coordinate data were not available, the coordinates of the capital of the province of origin were used*. T. turgidum* subsp. *durum* landraces are shown with circles, subsp. *dicoccon* with squares and subsp. *turgidum* with triangles

fastSTRUCTURE runs with 38,700 DArT markers divided the tetraploid wheat landraces into seven populations (K = 7) (Fig. 3a). All but one (BGE021775) of the 14 accessions belonging to subsp. *dicoccon* were grouped in Pop5, and all 37 of the subsp. *turgidum* accessions were clustered in Pop3. The landraces in both populations came mostly from the north of Spain (Fig. 3b). All of the 140 subsp. *durum* landraces except for one (BGE013103), which was classified into Pop3, were distributed among five populations (Pop1, Pop2, Pop4, Pop6 and Pop7), containing between 10 and 80 accessions (Additional file 1). Pop6 exhibited the highest number of accessions, showed the greatest degree of admixture and was the population with the most diverse eco-geographical origin (Fig. 3). However, some subsp. *durum* populations showed a narrower geographic distribution (Additional file 1). That is, the landraces in Pop1 originated mostly from eastern Spain, whereas those in Pop2 came from the south-western provinces. Pop4 included landraces from the South and East of Spain, and from the Canary Islands.

Genetic diversity parameters were calculated for the fastSTRUCTURE populations based on the SNP data (Table 2). The population with the highest genetic diversity value (Hs, Nei’s diversity index) was Pop6 (0.272), and the population with the lowest value was Pop2 (0.048). For the whole landrace collection, the D_est_ (Population differentiation index) value, a measure of population differentiation in collections with several populations, was 0.22. The F_ST_ values, which are related to genetic differentiation between populations, ranged from 0.743 (Pop2 vs Pop5) to 0.226 (Pop1 vs Pop6). Pop6 showed the least genetic differentiation from the rest of the populations, including those of subsp. *turgidum* (Pop3) and *dicoccon* (Pop5) (Table 2). When the diversity between the three subspecies was estimated, regardless of the structured populations, the *dicoccon* and *durum* landraces showed the highest value of genetic differentiation between the subspecies (F_ST_ = 0.42) whereas subsp. *turgidum* showed lower values compared to either *durum* and *dicoccon* (F_ST_ = 0.31 and 0.38, respectively).

**Table 2:** Genetic diversity within populations (Hs) and F_ST_ values between populations of *T. turgidum* landraces assessed with SNPs

Hs	0.186	0.048	0.253	0.089	0.169	0.272	0.104
F_ST_	**Pop1**	**Pop2**	**Pop3**	**Pop4**	**Pop5**	**Pop6**	**Pop7**
**Pop7**	0.476	0.709	0.474	0.653	0.692	0.238	–
**Pop6**	0.226	0.297	0.311	0.264	0.452		
**Pop5**	0.573	0.743	0.393	0.725			
**Pop4**	0.546	0.730	0.532				
**Pop3**	0.371	0.526					
**Pop2**	0.575						

The different populations names (Pop) are highlighted in bold

When we analyzed the distribution of Hs values across the genome, we detected some genomic diversity patterns that were population- specific (Additional file 6). For example, Pop2 and Pop7 presented a region of low diversity in the central part of chromosome 2A, while for chromosome 2B we only detected a region of low diversity in Pop5. On the other hand, a similar analysis contrasting the Hs values across the genome in the three durum wheat subspecies showed some common low diversity regions in *turgidum* and *dicoccon* (e.g., chromosomes 1A and 2A), while *durum* showed higher diversity values across the genome (Additional file 7).

**Fig. 4:**
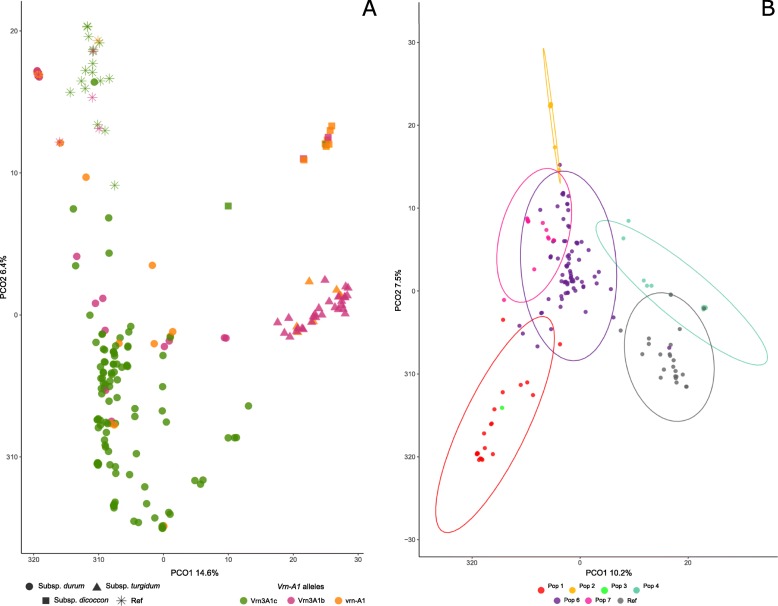
Cluster analysis of *T. turgidum* accessions using PCoA. Accessions from subsp. *durum* are shown with circles, subsp. *dicoccon* with squares, subsp. *turgidum* with triangles and the reference varieties with asterisks. **a** Graphical representation of PCo1 and PCo2 for the whole collection of durum wheat. Accessions are colored according to their *Vrn-A1* alleles. **b** Graphical representation of PCo1 and PCo2 for subsp. *durum* accessions, which are colored according to their STRUCTURE population assignment

We also explored the genomic structure of the durum wheat collection, including the landraces and reference varieties, through a principal coordinate analysis (PCoA) based on the 9324 filtered SNP markers. The first two principal coordinates explained 21% of the total variation. Three discrete groups corresponding to the three subspecies could be clearly identified (Fig. 4a). This was in agreement with the results obtained with fastSTRUCTURE where the three subspecies were grouped into different populations. A fourth group corresponding to the reference varieties also appeared to be clearly separated from the subsp. *durum* landraces. The subsp. *durum* accessions were differentiated from the others by PCo1, but the difference between *turgidum* and *dicoccon* was due to PCo2, demonstrating that different sets of markers are responsible of the genetic divergence among subspecies, as detected in the Hs analysis. Some landraces of subsp. *durum* were located close to subsp. *dicoccon* and *turgidum*. These landraces were from Pop6 and some of them (e.g., BGE019290) come from the North of Spain (Additional file 1).

We further investigated the allelic variability of a functional marker involved in wheat adaptability, the vernalization gene *Vrn-A1*, in relation to the population structure. Three different alleles were identified in the collection: the winter-type allele *vrn-A1,* and two alleles related to the spring growth habit, *Vrn-A1b* and *Vrn-A1c.* The representation of allelic variation in the PCoA showed that most of the accessions carrying the winter-type allele, *vrn-A1,* were grouped together and corresponded to *dicoccon* accessions (Fig. 4a). Most of the reference cultivars and subsp*. durum* accessions carried the *Vrn-A1c* allele, and almost all of the subsp. *turgidum* accessions carried the *Vrn-A1b* allele. When analyzed within the population structure, all but one *durum* accession from Pop2, Pop7, and Pop1 presented the *Vrn-A1c* allele, which was also identified in the 80% of the durum wheat landraces clustered in Pop 6. In Pop3 and Pop4, the most frequent allele was *Vrn-A1b*. According to passport data (see Additional file 1), the accessions with the winter-type allele *vrn-A1* came mostly from the North of Spain.

Allelic variation was also studied for the HMW-GS (High Molecular Weight Glutenin Subunits) loci *Glu-A1* and *Glu-B1*, which are related to wheat rheological properties, but no relationship with the population structure could be observed (data not shown).

As the subspecies was the main discriminant factor in the global PCoA, we decided to perform the analysis excluding the *turgidum* and *dicoccon* accessions to gain insight into the variability within subsp. *durum* (Fig. 4b). The populations identified in the previous analysis with DArTs (Fig. 3a) were similarly grouped in the SNP-based PCoA, with Pop6 again being the population showing the greatest dispersion due to its higher intrapopulation variability (Fig. 3a). The only *durum* accession clustered in Pop3 (BGE013103) appeared to be located close to the Pop1 landraces in this case (Fig. 4b). This local variety can be identified in Fig. 3a at the edge of Pop3, showing admixture with Pop1, which suggests that it could present a hybrid genotype between *durum* and *turgidum*.

Pop1, Pop2, and Pop7 were the *durum* populations that were most differentiated from the reference set. On the other hand, Pop4 was closest to the reference group. This population included old local varieties such as ‘Ledesma’ and ‘Lebrija’, obtained from crosses between ‘Senatore Capelli’ and Spanish landraces. One landrace from Pop6 of subsp. *durum* (BGE026954) was grouped together with the reference varieties. This accession, collected at the end of the 1990, is characterized by early-maturity and short plants [22], which suggests that it is probably not a true landrace.

### Genetic structure of the bread wheat collection

**Fig. 5:**
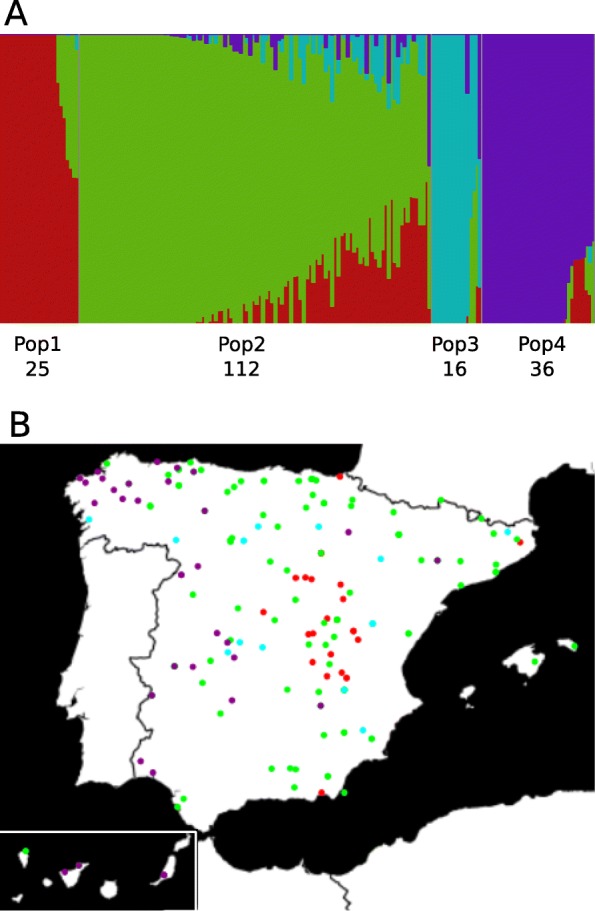
**a** *T. aestivum* STRUCTURE plot based on DArT markers. The number below the Pop indicates the number of accessions clustered in each population. **b** Collection sites of the different *T. aestivum* accessions, colored by their STRUCTURE population assignment. When GPS coordinate data were not available, the coordinate of the capital of the province of origin were used

fastSTRUCTURE runs with 44,241 DArT markers divided the hexaploid wheat landrace accessions into four populations (K = 4). Compared to durum wheat landraces, a higher level of admixture was detected in the bread wheat populations, especially within Pop2, which was the largest population, containing 112 accessions (Fig. 5a, Additional file 1). The landraces from Pop1 came from central Spain, and the landraces from Pop4 came from the west, including the Canary Islands. Pop2 and Pop3 showed more diverse eco-geographical origins (Fig. 5b, Additional file 1).

As shown in Table 3, the population with the highest Hs was Pop2 (0.277), and the population with the lowest value was Pop3 (0.101). In the whole landrace collection, the D_est_ value was 0.17, which was lower than the differentiation found in the durum collection (0.22). The F_ST_ values between populations ranged from 0.169 (Pop1 vs Pop2) to 0.573 (Pop3 vs Pop4). According to the F_ST_ values, Pop4 was the most differentiated population (Table 3). Regarding the Hs distribution across the genome, we detected some low-diversity regions specific to certain populations (Additional file 8). For instance, Pop3 and Pop4 showed low-diversity regions on chromosomes 1A and 7A, while Pop1 and Pop3 showed low diversity on chromosomes 3A and 2B. This suggests that different genomic regions are responsible for the divergence among populations.

**Table 3:** Genetic diversity within populations (Hs) and F_ST_ values between populations of *T. aestivum* landraces assessed with SNPs

Hs	0.176	0.277	0.101	0.188
F_ST_	**Pop1**	**Pop2**	**Pop3**	**Pop4**
**Pop4**	0.506	0.346	0.573	–
**Pop3**	0.483	0.265		
**Pop2**	0.169			

The different populations names (Pop) are highlighted in bold

**Fig. 6:**
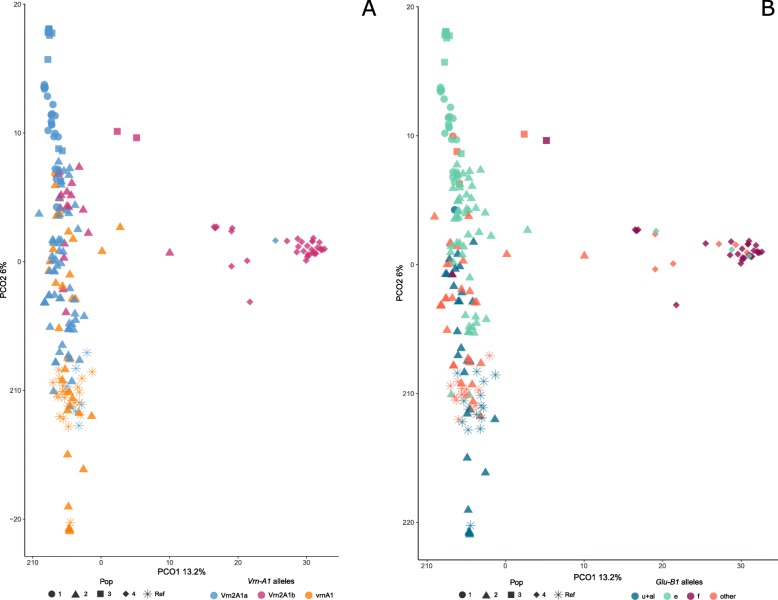
Cluster analysis of *T. aestivum* accessions using PCoA. Accessions from STRUCTURE Pop1 are shown with circles, Pop2 with triangles, Pop3 with squares, Pop 4 with rhombi, and the reference varieties with asterisks. **a** Graphical representation of PCo1 and PCo2 for the whole collection of bread wheat. Accessions are colored according to their *Vrn-A1* allele. **b** Accessions are colored according to their *Glu-B1* allele

The relationships among the bread wheat accessions were also assessed by PCoA, based on 8238 SNPs in the whole bread wheat collection. The total amount of genetic variation explained by the first two principal coordinates was 19.2%. The first two coordinates clearly separated the Pop4 (by PCo1), which formed the most distant group, from the other three populations, which appeared to be distributed along PCo2 (Fig. 6a). Some degree of overlap was shown between Pop2, distributed along PCo2, and both Pop1 and Pop3, located at the upper extreme of PCo2. These results are in agreement with the higher degree of admixture in Pop2 revealed by fastSTRUCTURE analysis (Fig. 5a). The reference varieties were located within a quite limited space but overlapped with some Pop2 landraces.

As in durum wheat, we investigated the allelic variability of the *Vrn-A1* gene in relation to the bread wheat population structure. Three different alleles were identified in the collection: the winter-type allele *vrn-A1* and two alleles that are generally related to a spring growth habit, *Vrn-A1a* and *Vrn-A1b.* The accessions from Pop4 were characterized by almost exclusively presenting the *Vrn-A1b* allele. The accessions presenting the other two alleles could be differentiated along the PCo2 axis, whose upper portion corresponded to spring-type landraces, whereas the reference varieties and winter-type landraces were included in the lower portion (Fig. 6a).

The representation of *Glu-B1* alleles specific to Iberian landraces in the PCoA showed that the accessions that carried the *Glu-B1f* allele (HMW-GS 13 + 16) were clustered on the right side of the PCoA and corresponded to most of the Pop4 samples. Again, an interesting tendency could be observed along the PCo2 axis: accessions with the *Glu-B1e* allele (HMW-GS 20x + 20y) appeared to be grouped in the upper portion, while the lower portion corresponded to accessions with *Glu-B1u* or *Glu-B1al* alleles (HMW-GS 7 + 8 or 7OE+ 8) (Fig. 6b). The allelic variation was also studied for HMW-GS loci *Glu-A1* and *Glu-D1*, but no relationship with the population structure could be observed (data not shown).

The overlap between the landraces and reference varieties was more remarkable in bread wheat than in durum wheat. In fact, the reference varieties fully overlapped with the Pop2 accessions. Some of the landraces located closer to the reference varieties were collected in the 1990s (e.g., BGE025410), and according to their early flowering and lower height phenotypes (http://webx.inia.es/web_coleccionescrf/CaracterizacionCRFeng.asp), they may not be real landraces.

### Divergence between landraces and reference varieties

As noted above, PCoA showed clear genetic divergence between the landraces and reference varieties of durum wheat, whereas such divergence was not as evident in bread wheat, in which the landraces and reference varieties overlapped (Figs. 4 and 6). Regarding the overall genetic diversity, the landraces showed higher diversity than the reference varieties in both species (Table 4), with the difference being greater in durum wheat.

**Table 4:** Overall genetic diversity (Hs) and number of monomorphic SNP markers in the set of reference varieties compared to landraces

	*Triticum turgidum*		*Triticum aestivum*	
	Reference	Landraces	Reference	Landraces
Hs	0.196	0.323	0.250	0.300
Monomorphic markers	3791	24	1771	2

The divergence between the reference varieties and landraces varied among the different populations identified by fastSTRUCTURE. The durum wheat reference varieties showed the highest differentiation from the landraces in Pop5 (*dicoccon*) and Pop2 (*durum*), and the lowest differentiation from the *durum* landraces in Pop4 and Pop6 (F_ST_ values of 0.586, 0.555, 0.372 and 0.214, respectively). In bread wheat, the reference varieties showed the highest and lowest differentiation from the landraces in Pop4 and Pop2, respectively (F_ST_ values of 0.416 and 0.08, respectively).

To analyze the degree of allele fixation in the reference varieties, we studied the presence of monomorphic markers. Approximately, 40% of the SNP markers were fixed in the durum wheat reference varieties, and the number of monomorphic markers (3791) was comparable to that found in subsp. *dicoccon* and *turgidum* (4079 and 2119 respectively) and much higher than that found in subsp. *durum* (478 fixed markers). In the bread wheat reference accessions, the number of fixed SNP markers was 1771 (21%) clearly lower than the number obtained in durum wheat (Table 4).

**Fig. 7:**
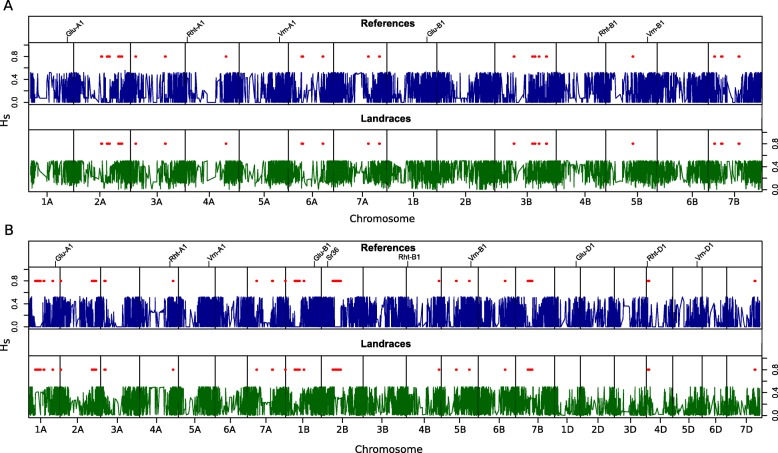
Genetic diversity (Hs) distribution across the genome in the reference varieties and landraces. **a** *T. durum*. **b** *T. aestivum*. Fixed regions in the reference materials are indicated by red bars. The position of ten key genes in the genome is indicated

We also analyzed the Hs distribution across the genome in the landraces and reference varieties (Fig. 7). We called low-diversity regions in the reference varieties with respect to the landraces, as they might be regions fixed by breeding efforts. For this particular analysis, we employed a different set of markers, as described in Methods section, which include only SNPs located in the reference genomes. In durum wheat, we detected 20 of these genomic regions (Fig. 7a). Chromosomes 1A, 1B, 2B, 4B, 5A and 6B did not present low-diversity regions, while the rest of chromosomes presented at least one fixed region. The largest wheat chromosome (3B) presented the greatest number of low diversity regions, with 5 regions spanning a total of 39 Mb. In turn, chromosome 2A presented the widest region of low diversity (37 Mb). In summary, the identified low-diversity regions spanned 218 MB. In bread wheat, a similar number [24] of lower-diversity regions were called (Fig. 7b). Interestingly, the D genome included only two low-diversity regions. In the A genome, we detected 12 such regions located on chromosomes 1A, 2A, 3A, 4A and 7A, while in the B genome, we observed low-diversity regions on all the chromosomes except for 3B. The widest fixed region was located on chromosome 2B, spanning 121 Mb, and chromosome 2A contained the greatest number of regions, with 4 regions. In bread wheat, the low-diversity regions spanned a total of 601 MB and included the semidwarfing gene *Rht-D1*, a key gene introduced in wheat cultivars during the 20th that is located on chromosome 4D, and *Vrn-B1*, located on 5B and related with growth habit.

## Discussion

Modern bread and durum wheat varieties have resulted from breeding programs that have mainly been focused on achieving high yields in conventional agricultural systems. The unpredictable effects on crop performance under future climate scenarios and the increasing concern for the environmental consequences of crop management practices, which are commonly based on excessive irrigation and fertilizer application, are reorienting breeders’ aims toward the development of new wheat cultivars that can maintain high yields under uncertain climatic conditions and in a more sustainable manner. For this purpose, genetic resources maintained in wheat genebanks, which have generally been underused, need to be thoroughly characterized so that the useful genetic variation that is present is transferred to modern elite gene pools. Several recent international initiatives are characterizing local wheat germplasm collections (www.seedsofdiscovery.org; www.divsek.org). However, Spanish landraces, which are locally adapted to a wide diversity of eco-climatic conditions and mostly cultivated under rainfed low-input management systems, are absent or only represented by a small number of accessions in these studies. We decided to explore the genetic diversity of these valuable materials, by performing an analysis at the genomic level of 380 selected landraces representing the genetic variability of the wheat collection maintained at the Spanish Plant Genetic Resources Center, which is composed of more than 1600 accessions.

### Wheat high-throughput genotyping

This is the first report of the high-throughput genotyping by GBS of Spanish wheat germplasm. The GBS method has the potential to provide robust in-depth genomic diversity estimates. Moreover, this approach may reveal new alleles that might present high value for prebreeding [30–32]. The DArTseq-GBS methodology has been successfully applied in wheat species, in which a standard GBS approach, requiring genome resequencing, is still challenging due to the extremely large complex genome [10].

In our study, 50 K SNP and 100 K DArT markers were analyzed in each of the wheat species, from which we were able to select approximately 10 K SNP and 40 K DArT high quality markers. The availability of the reference bread wheat genome [4] and durum wheat genome [5] allowed the marker location to be performed in both species. The results showed that markers were distributed throughout the genome, although D genome markers were markedly less abundant than A and B genome markers in bread wheat. Several previous studies have shown the reduced diversity of the wheat D genome, which has been explained by the close genetic distance between the *Ae. tauschii* parents involved in the formation of hexaploid wheat (e.g. [33, 34]). Among homoeologous sets, the group 4 chromosomes were the least covered, especially 4B and 4D. No satisfactory hypothesis has yet been proposed to explain the relative scarcity of markers consistently mapped on 4B compared to 4A [35, 36], despite the latter being involved in several structural rearrangements in wheat [37].

The informativeness of the markers was assessed from their PIC values, and the distribution and average PIC value were found to be comparable to those previously reported in wheat [20, 38–41].

### Population structure of Spanish wheat landraces

When a large germplasm collection is used for the identification of useful alleles or traits, knowledge of its genetic structure is highly recommended to optimize the search approach, which can then be focused on a smaller sample. The study of population structure is also important for genomic studies, as it is a mandatory prerequisite for successfully performing further GWAS analyses. In our case, since future GWAS analyses will be based on the high-quality SNPs identified herein, we decided to assess the structure with a different set of markers, DArTs. Population structure was assessed with the fastSTRUCTURE algorithm, which was developed by the authors of the classical STRUCTURE software but shows faster runs and provides comparable ancestry estimates and prediction accuracies [42]. In both species, the analysis of population structure was complemented with PCoA performed with the SNP dataset. As expected, the results were in close agreement, but some additional information about the subgroups and their relationship to phenotypic traits could be extracted.

#### Durum wheat landraces

The Spanish durum wheat (*Triticum turgidum* L.) landraces belong to three main interfertile subspecies (*dicoccon*, *turgidum* and *durum*). The subsp. *dicoccon*, also known as emmer wheat, is a hulled wheat that is only grown in the North of Spain and represents the feral situation of this crop. Subsp. *durum* is the most widely cultivated and well adapted to the dry-summer conditions of the South. Subsp. *turgidum*, which is less common and grown in colder areas than *durum*, mostly consists of winter wheat [43]. In a previous study, SSRs (Single Sequence Repeats) were used to assess the genetic structure of the collection of durum wheat landraces analyzed here and 9 populations were established. Some of the populations included more than one subspecies and several genotypes could not be classified into any population [27]. In the present work, we identified seven populations, all of which were composed of landraces from one subspecies with only two exceptions (Fig. 3). The discrepancies between this and the earlier work can be explained by the different types and numbers of markers employed for assessing the population structure in both studies (40 K DArTs vs 39 SSRs).

Analyses comprising other durum wheat materials have not been able to separate the *durum* and *turgidum* subspecies [44, 45]. However, we clearly differentiated the three subspecies. This demonstrates the analytical power of DArT and SNP markers for taxonomical identification and supports the classification of Mac Key [46, 47], with *turgidum* and *durum* as separate subtaxa. In our study, subsp. *dicocco*n was closer to *turgidum* than to *durum* (see F_ST_ values, Table 2), indicating that the two subspecies share a common allele pool. As the two subspecies were grown in similar environmental conditions, this closeness might be due to similar selective pressures during local adaptation. The analysis also allowed the identification of accessions that could represent admixtures between subspecies, such as BGE021775, a *dicoccon* landrace located closer to *durum*, or BGE04564, a *durum* landrace grouped with the *turgidum* landraces. It has been reported that such admixture is not unusual in ancient local forms of durum wheat [47].

Vernalization genes are the main determinants of the growth habit (i.e., winter or spring) in temperate cereals, and by affecting the vegetative to reproductive transition, these genes are involved in the ability of wheat plants to adapt to a wide range of environments [48]. The evolution of spring-habit cultivars from winter-habit accessions played a key role in the postdomestication spread of wheat. However, studies on the major vernalization gene *VRN1* have been mostly limited to hexaploid wheat species and very few reports from tetraploid species can be found in the literature [48, 49]. None of the durum wheat accessions characterized here showed the *Vrn-A1a* allele described in spring-habit hexaploid wheat varieties, which is in accordance with what has been found in other studies [50, 51]. All of the available data seem to indicate that this allele appeared during wheat evolution after the last polyploidization event. The *Vrn-A1c* allele has been described as the most frequent determinant of a spring habit in tetraploid wheat varieties [50, 52, 53] but has been described as rare in emmer wheat varieties [44]. In our study, 78% of the *durum* accessions presented this allele, but almost all *turgidum* accessions (83%) carried *Vrn-A1b*, and 7 out of the 11 *dicoccon* landraces characterized for *Vrn-A1* carried the winter *vrn-A1* allele (Fig. 4). The presence of the spring-habit associated alleles *Vrn-A1b* and *Vrn-A1c* in the remaining four *dicoccon* accessions is remarkable. Emmer wheat varieties are traditionally cultivated in cool mountainous regions, where vernalization seems to be an unavoidable requirement. It may explain why few Spanish spring emmer wheat varieties have actually been described [43]. The spring type might have evolved from previous winter types as an adaptation to warmer conditions. Under the predicted climate change scenario, temperature warming may prevent the fulfilment of the requirements for vernalization in current temperate zones, thus having a negative global impact on winter wheat yields. The identification of genotypes with reduced vernalization requirements among germplasm adapted to cool zones could therefore be relevant for improving adaptability to changing eco-climatic conditions.

Within our collection, a restricted geographic distribution exists for the Spanish landraces belonging to the less represented subspecies *dicoccon* and, to some extent, for *turgidum* landraces. The clustering of these two groups of accessions, each in a single population, may reflect similar environmental conditions in their respective geographic origins. The landraces of subsp. *durum*, which were structured into five distinct populations, showed higher variability and greater complexity, including different phylogenetic groups [27]. The identified clusters seemed to be influenced by the accessions’ origin relatively little, although some geographic areas were predominant in some of the populations. The gene flow between regions via germplasm exchanges and local preferences towards a given agrotype might be as significant as ecological conditions in determining the distribution of genetic diversity in this subspecies in Spain. The exchange of seeds by farmers has been noted as one of the likely explanations for the low or absent influence of geographic origin on the genetic structure of durum wheat landraces in Iran, the Central Fertile Crescent and Ethiopia [20, 54, 55].

#### Bread wheat landraces

The great majority of the Spanish bread wheat landraces conserved at CRF-INIA belong to *Triticum aestivum subsp. vulgare*, and our bread wheat set was therefore composed exclusively of this subspecies. The population stratification of the bread wheat panel identified four groups of landraces with high divergence according to the obtained F_ST_ values. This clustering reflected the geographic origin of the accessions better than in the subsp. *durum.* The genetic differentiation estimated with D_est_ was lower in the bread landrace collection than in the whole durum wheat collection, in agreement with the lower level of stratification observed. Few studies have simultaneously addressed the variability of hexaploid and tetraploid wheat varieties but higher genetic diversity in durum than in bread wheat has been previously reported in landraces from other countries (e.g., [56]). Several studies support the occurrence of a limited number of independent crosses between the diploid and tetraploid progenitors of *T. aestivum*, where the resulting loss of diversity during the initial polyploidization step presumably caused a severe population bottleneck in hexaploid bread wheat [33, 57].

One of the four groups detected (Pop4) was clearly more genetically distant. This group included landraces from western Spain, where there is a prevalence of acidic or neutral soils [58]. Most of the accessions from this population show spring growth habit and carry the *f* allele at the *Glu-B1* locus. The *Glu-B1f* allele presents a low frequency in worldwide collections but has been previously described as being characteristic of Iberian landraces, which is also the case for the *Glu-B1e* allele [59]. The latter was predominant in Pop1 and Pop3, and was also present in some landraces of Pop2 that were closely grouped by PCoA. The *Glu-B1e* allele is related to poor rheological properties in bread wheat, but the *f* allele has been associated with good dough quality [60]. The presence of this variant in a discrete group of more differentiated landraces supports their common origin.

In our study, the winter-type allele *vrn-A1* was the most common *Vrn-A1* allele found in the *T. aestivum* reference cultivars (22 out of 29 varieties). However, the most frequent of these alleles within the bread wheat landraces was the *Vrn-A1a* allele, which was absent in the durum wheat. This *Vrn-A1* allele, which results in complete insensitivity to vernalization, is recognized as the spring-habit allele with the greatest effect among all such alleles described [61]. Even if the growth habit shows just a limited impact on genetic differentiation, spring and winter bread wheat accessions are frequently separated by discriminant analysis of principal components [62, 63]. Two discrete groups regarding growth habit were not clearly defined in our PCoA, but the allelic variability of *Vrn-A1* showed some relationship to population structure and a somewhat biased tendency along the second PCo axis (Fig. 6a). Regarding this matter, it must be kept in mind that the vernalization response is a complex process under polygenic control [64]. *Vrn-A1* has been described as the main genotypic determinant of the vernalization requirements of temperate crops, but there are other genes, such as *Vrn-B1* and *Vrn-D1*, whose allelic variability has not yet been characterized in these Spanish wheat landrace collections.

This is the first time that the bread wheat resources maintained in the CRF-INIA Spanish national genebank have been characterized at the genetic level and compared to durum wheat accessions. This deep knowledge of their genetic structure represents the starting point for the development of a core collection of Spanish *T. aestivum* wheat varieties, which will allow the efficient management and use of this valuable gene pool.

### Relationship between landraces and modern cultivars

In diversity studies, wheat landraces usually cluster in a separate group from elite cultivars [54, 55, 63, 65] but some degree of mixture has also been found [39, 62]. Concerning the durum wheat materials examined here, the great genetic divergence between the bulk of the landraces and the reference set is remarkable (Fig. 4a). Moreover, some of the *durum* landraces were located closer to *turgidum* and *dicoccon* accessions than to the reference varieties (which all belong to subsp. *durum*). However, high relatedness to the reference varieties was detected for a reduced group of *durum* landraces. This group included ‘Caravaca’ (BGE002869), a Spanish landrace used by CIMMYT in the development of some modern cultivars (see the Genetic Resources Information System for Wheat and Triticale of CIMMYT at http://www.wheatpedigree.net/). It can also be noted that other studies have reported a close relationship between Spanish and North African landraces [39, 44] and that the reference set included two old cultivars that were commonly cultivated in Spain in the past (‘Senatore Capelli’ and ‘Bidi-17’), both of which exhibit a North African origin. Nevertheless, our results supported little involvement of Spanish landraces in the development of the modern durum wheat varieties grown in Spain at present.

In bread wheat, the situation was quite different, and the mixing between the landraces and reference varieties was much higher, especially for some accessions in Pop2 (Fig. 6). It is likely that some of these accessions are not true local landraces but, rather, old improved cultivars that were wrongly classified. The clustering of the landraces and reference varieties could also indicate a pedigree relationship. Hence, it is possible that some of the landraces characterized in our study were among the unidentified “Mediterranean” local varieties utilized by the early breeders as starting material to develop pure lines that were further involved in cross-breeding (see http://www.wheatpedigree.net/). Some other varieties such as ‘Richela Blanca’, ‘Montnegre’ or ‘Ardito’, are related to old Italian material [43, 66], which provides another feasible genealogical link between the sets of landraces and reference bread wheat materials analyzed.

The overall genetic diversity of the reference cultivars was much lower than that of the landraces in both species (Table 2; Figs. 4 and 6). Genomic regions showing patterns of variation that differ between landraces and varieties can aid in the identification of loci under selection during crop improvement, which will help to better target future breeding efforts [67]. Our analysis allowed the identification of several such genomic regions by studying the distribution of genetic diversity across the reference genomes (Fig. 7). The number of regions that have presumably been fixed by selective breeding was higher in bread wheat that in durum wheat. As expected, some of the chromosomes including fixed regions harbored genes related to agronomically important phenotypes, such as *Rht-D1*, associated with dwarf phenotype, and *Vrn-B1*, associated with vernalization response, on bread wheat chromosomes 4D and 5B, respectively [68, 69]. In turn, the fixed region on bread wheat chromosome 1A may be related to the presence in this chromosome of major determinants of bread quality, such as the HMW-GS encoding the *Glu-1A* locus. Coupling this analysis with future GWA studies will help to identify the traits underlying each of the fixed regions detected in this work.

## Conclusions

The replacement of local landraces by high-yielding wheat varieties that began at the time of the Green Revolution has led to a loss of genetic variation in crop wheat varieties. This depletion has now encouraged the use of genetic resources in wheat breeding programs, but the genetic variability of these resources needs to be exhaustively characterized for their efficient use. The present study successfully used DArTseq technology for evaluating the diversity within and between two landrace collections of bread and durum wheat and for assessing the genetic relationships between each of these collections and a reference set of modern wheat cultivars. The study of genome-wide diversity provides a resource for the design of high-power GWAS experiments, which will help to achieve the overarching goal of improving wheat for cultivation in different environments, ecosystems and stress situations. The collections of Spanish landraces characterized in the present study were clearly clustered into different groups, representing different gene pools capable of providing different sources of genes for plant breeding. The investigated panel of genotypes showed an outstanding degree of diversity compared to the reference counterparts. It therefore clearly represents a strategic platform and a valuable genetic resource that must be further studied to ensure not only its efficient conservation and management but also its useful exploitation in breeding programs.

## Methods

### Plant material

The plant material analyzed in the present study comprised 432 selected accessions (detailed information is presented in Additional file 1). This sample included 191 durum and 189 bread wheat landraces and old local cultivars (hereafter referred to as landraces), representing different ecological and geographical areas of Spain [27, 70]. Among the durum wheat landraces, 140, 37 and 14 landraces corresponded to the subspecies *durum*, *turgidum* and *dicoccon*, respectively, while all the bread wheat landraces belonged to the subspecies *vulgare*. All these accessions were homozygous lines derived from genebank accessions provided by CRF-INIA. Additionally, the study included a set of 23 improved varieties of durum wheat and 29 improved varieties of bread wheat (hereafter referred to as reference varieties), which comprised the cultivars most widely grown in Spain during the last 50 years plus some varieties widely used in wheat research, such as tetraploid ‘Langdon’ and hexaploid ‘Chinese Spring’.

### DNA isolation and genotyping analysis

For each accession, genomic DNA was isolated from the young leaves of a single plant using the CTAB method [71]. Samples were genotyped using DArTseq GBS technology at Diversity Arrays Technology Pvt., Ltd. (Canberra, Australia) for the durum wheat accessions and SAGA (Genetic Analysis Service for Agriculture, Mexico City, Mexico) for the bread wheat accessions [72, 73].

A complexity reduction method including two enzymes (PstI and HpaII) was used to create a genome representation of the set of samples. PstI-RE site specific adapter was tagged with 96 different barcodes enabling multiplexing a 96-well microtiter plate with equimolar amounts of amplification products in order to run within a single lane on Illumina HiSeq2500 instrument (Illumina Inc., San Diego, CA). The successful amplified fragments were sequenced up to 77 bases, generating approximately 500,000 unique reads per sample. Thereafter the FASTQ files (full reads of 77 bp) were quality filtered using a Phred quality score of 30, which represent a 90% of base call accuracy for at least 50% of the bases. More stringent filtering was also performed on barcode sequences using a Phred quality score of 10, which represent 99.9% of base call accuracy for at least 75% of the bases. A proprietary analytical pipeline developed by DArT P/L was used to generate allele calls for SNP and DArT markers.

After this process the genotyping services provided two different sets of markers. The DArT markers were scored as binary data (0/1) indicating the presence or absence of a marker in each accession, and the SNP markers were scored as 0/1/2 indicating the presence of the reference allele in homozygosity, the alternative allele in homozygosity or a heterozygous genotype, respectively. The raw data are available upon request to the corresponding author. To locate the markers in the durum and bread wheat reference genomes, the markers sequences were subjected to BLAST searches against the currently available *Triticum aestivum* genome IWGSC Refseq v1.0 [4] for bread wheat markers and *Triticum turgidum* genome Svevo v1.0 [5] for durum wheat markers. A marker was located according to the following criteria BLAST E-value <5e-10 and sequence identity > 90%.

For comparison with the population structure based on GBS-DArTseq markers, we investigated the allelic variability of functional markers in the *Vrn-A1* gene, one of the most determinant loci involved in the transition from vegetative to reproductive growth [74, 75]. It has been described that carrying a dominant allele at the *Vrn-A1* locus is sufficient to confer a spring growth habit [53]. Three alleles (*Vrn-A1a*, *Vrn-A1b* and *Vrn-A1c*) were characterized by PCR according to [51, 52, 68] and following the protocols described at https://maswheat.ucdavis.edu/protocols/Vrn/index.htm.

Additionally, the panel of accessions was genotyped for *Glu-1* homoeoloci. These complex loci encode the HMW-GS, which are the major determinants of dough quality in wheat. For this purpose, endosperm proteins were extracted from single seeds and fractionated via sodium dodecyl sulfate polyacrylamide gel electrophoresis (12% polyacrylamide gels) according to Payne et al. [7^6^]. HMW-GS allele classification was performed according to the Catalogue of Gene Symbols for Wheat 2013 [77].

### Data analysis

Prior to any further analysis, the set of SNP and DART markers was filtered employing homemade R scripts [78], which are available upon request to the corresponding author. For DART markers we selected high quality markers following a step-by-step filtering strategy. First, when several markers presented the same allelic profile, all of the markers but the one with the least missing data were removed. Then, the markers that presented more than 10% missing data or were monomorphic (Minimum Allele Frequency, MAF < 0.05) were excluded.

For the SNP markers, prior to any filtering step we analyzed the presence of heterozygous genotypes. As we have previously described, genotyping was conducted on homozygous lines and thus we did not expect any heterozygous genotypes. When the genotypic values for a marker was only 0 and 2, we considered the heterozygous calling [2] to be an error caused by the presence of the SNP marker flanking sequence in homoeologous genomes. In this case, the genotypes scored as 2 were recoded as 1. The same procedure was followed when only genotypic values of 1 and 2 were present in a marker, but in those cases, genotypes scored as 2 were recoded as 0. Finally, when the genotypic values for a marker included 0, 1 and 2 we recoded the genotypes scored as 2 as missing data. After this analysis we selected high quality SNP markers following the filtering strategy described for the DART markers. First, when several markers presented the same allelic profile, all but the one with the less missing data were removed. Then, markers with more than 10% missing data or monomorphic (MAF < 0.05) were excluded.

The genetic substructure within the durum and bread wheat landrace collections was investigated using the fastSTRUCTURE algorithm [42] and the DArT marker dataset (including all the available DArTs after filtering, located or not in the reference genomes). Default parameters and K values from 1 to 15 were tested. The appropriate number of components that explained the structure in the dataset was determined using the chooseK.py function [42]. The results for the identified optimal values of K were visualized using DISTRUCT [79]. Individual accessions were assigned to the population with the highest proportional membership.

The genetic similarity based on the SNP data within the full sets of accessions (landraces plus reference varieties) was analyzed by principal coordinates analysis (PCoA) using the gl.pcoa function from the dartR R package [80] (including all the available SNPs after filtering, located or not in the reference genomes).

The gene diversity (Hs) within populations, landraces and reference varieties was calculated based on the SNP dataset according to [81] with the basic.stats function from the hierfstat R package [82]. D_est_, a measure of population differentiation in collections with several populations, was calculated as defined by [83] with this same function. The genetic differentiation between populations was analyzed by estimating the pairwise fixation index (Fst) according to [84] with the stamppFst function from R the StAMPP package [85] using the SNP dataset (including all the available SNPs after filtering, located or not in the reference genomes).

Fixed genomic regions in the reference varieties were identified by performing a scan of the Hs values along the different chromosomes. Hs was estimated as described previously [81]. However, for this analysis we obtained a new SNP dataset by avoiding the first filtering step, thus when several markers presented the same allelic profile we kept all of them and only filter out the markers that presented more than 10% missing data or were monomorphic (MAF < 0.05). Finally, for this particular analysis only markers with known location in the reference genomes were employed. A region was considered to be “fixed” when it contained at least 5 consecutive markers with an Hs equal to 0 in the reference varieties and at least 5 markers with an Hs > 0.1 in the landraces, and spanned more than 5 Mb.

## Supplementary information

**Additional file 1. **Excel file with the description of the accessions analyzed, passport data, population according to fastSTRUCTURE analysis, HMW-GS and *VRN-A1* alleles. Sheet 1. Durum wheat. Sheet 2. Bread wheat. Sheet 3. Reference set.

**Additional file 2. **File with the *T. durum* accession raw DArT genotyping results.

**Additional file 3. **File with the *T. durum* accession raw SNP genotyping results.

**Additional file 4. **File with the *T. aestivum* accession raw DArT genotyping results.

**Additional file 5. **File with the *T. aestivum* accession raw SNP genotyping results.

**Additional file 6.** Figure showing the genetic diversity (Hs) distribution across the A and B genomes in the different durum wheat populations identified with fastStructure.

**Additional file 7.** Figure showing the genetic diversity (Hs) distribution across the A and B genomes in the different durum wheat subspecies.

**Additional file 8.** Figure showing the genetic diversity (Hs) distribution across the A, B and D genomes in the different bread wheat populations identified with fastStructure.

## Data Availability

Plant material and raw data are available upon request to the corresponding author.

## Abbreviations

CIMMYT: Centro internacional de Mejoramiento de Maíz Y Trigo
CRF-INIA: National Plant Genetic Resources Center
DArT: Diversity Array Technology
DArTseq: Diversity Array Technology sequence
D_est_: Jost’s Population Differentiation index
F_ST_: Wright’s Fixation index
GBS: Genotyping By Sequencing
GWAS: Genome Wide Association Studies
HMW-GS: High Molecular Weight Glutenin Subunits
Hs: Nei’s diversity index
MAF: Minimum Allele Frequency
MAS: Marker assisted selection
PCoA: Principal coordinates analysis
PIC: Polymorphic Information Content
SNP: Single nucleotide polymorphism
SSRs: Single Sequence Repeats
