# Supplementary material for: Genomic analysis of Spanish wheat landraces reveals their variability and potential for breeding
Source: BMC Genomics. 2020 Feb 4;21:122. doi: 10.1186/s12864-020-6536-x (PMC7001277; doi:10.1186/s12864-020-6536-x)
Supplement: Supplementary file 8 — Additional file 8. Figure showing the genetic diversity (Hs) distribution across the A, B and D genomes in the different bread wheat populations identified with fastStructure. [file 12864_2020_6536_MOESM8_ESM.pdf]

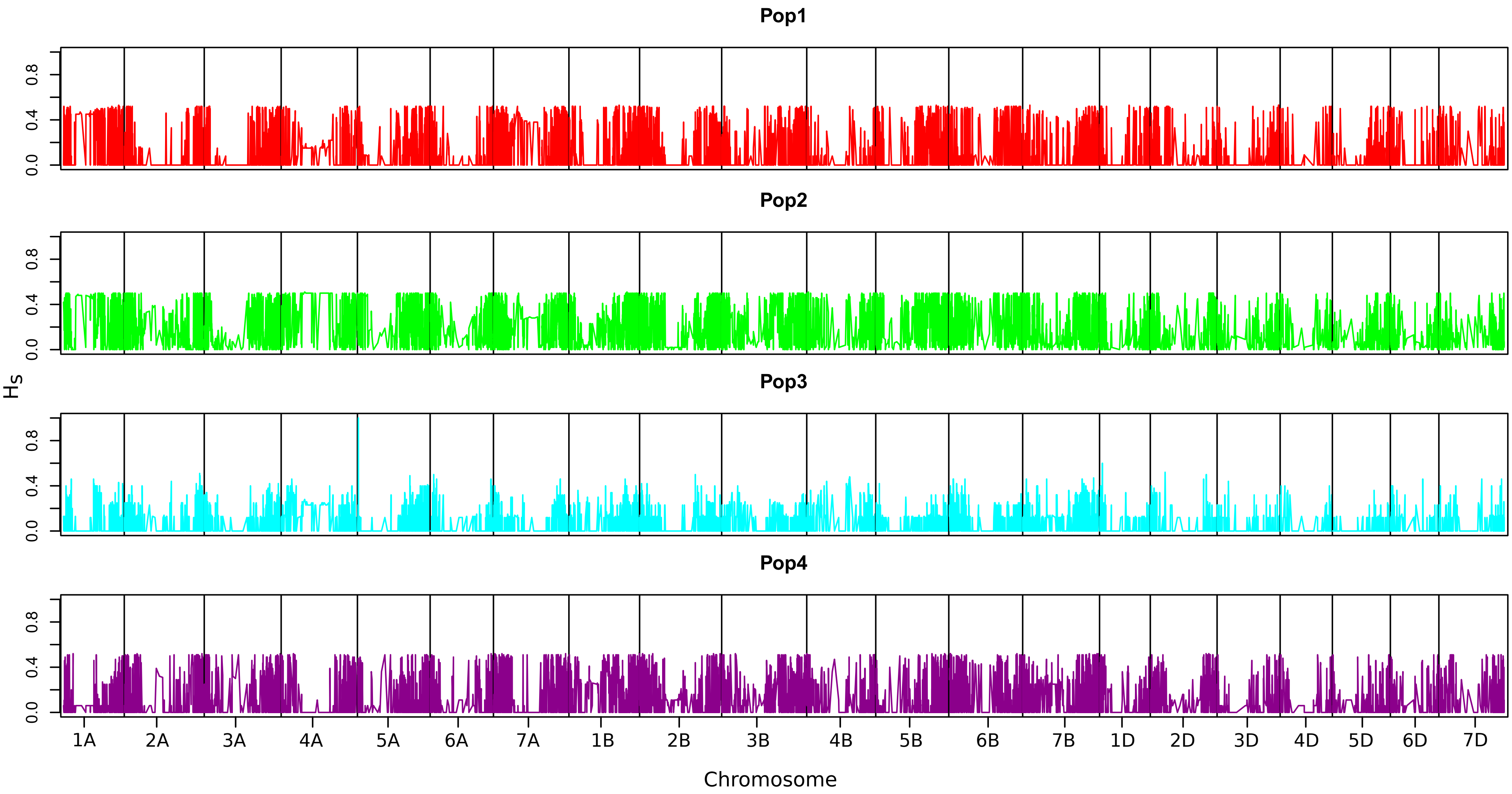

Additional file 8. Figure showing the genetic diversity (H_S) distribution across the A, B and D genomes in the different bread wheat populations identified with fastStructure.
